# An endogenous PI3K interactome promoting astrocyte-mediated neuroprotection identifies a novel association with RNA-binding protein ZC3H14

**DOI:** 10.1074/jbc.RA120.015389

**Published:** 2020-12-03

**Authors:** Samih Alqawlaq, Izhar Livne-Bar, Declan Williams, Joseph D'Ercole, Sara W. Leung, Darren Chan, Alessandra Tuccitto, Alessandro Datti, Jeffrey L. Wrana, Anita H. Corbett, Gerold Schmitt-Ulms, Jeremy M. Sivak

**Affiliations:** 1 Department of Vision Science, Krembil Research Institute, University Health Network, Toronto, Ontario, Canada; 2 Department of Ophthalmology and Vision Science, University of Toronto School of Medicine, Toronto, Ontario, Canada; 3 Tanz Centre for Research in Neurodegenerative Diseases, University of Toronto, Ontario, Canada; 4 Department of Biology, Emory University, Atlanta, Georgia, USA; 5 Lunenfeld Tanenbaum Research Institute, Mount Sinai Hospital, Toronto, Ontario, Canada

**Keywords:** astrocyte, neuroprotection, interactome, ZC3h14, PI3K, PDGF, ACM, astrocyte conditioned media, CFM, cell-free media, PDGFRA, platelet-derived growth factor receptor A, PI3K, phosphoinositide 3-kinase, PLA, proximity ligation assay, poly(A), polyadenosine, PSM, peptide-to-spectrum matches, RBP, RNA-binding protein, RGC, retinal ganglion cell, THOC1, THO complex protein 1, ZC3H14, zinc finger CCCH-type containing 14

## Abstract

Astrocytes can support neuronal survival through a range of secreted signals that protect against neurotoxicity, oxidative stress, and apoptotic cascades. Thus, analyzing the effects of the astrocyte secretome may provide valuable insight into these neuroprotective mechanisms. Previously, we characterized a potent neuroprotective activity mediated by retinal astrocyte conditioned media (ACM) on retinal and cortical neurons in metabolic stress models. However, the molecular mechanism underlying this complex activity in neuronal cells has remained unclear. Here, a chemical genetics screen of kinase inhibitors revealed phosphoinositide 3-kinase (PI3K) as a central player transducing ACM-mediated neuroprotection. To identify additional proteins contributing to the protective cascade, endogenous PI3K was immunoprecipitated from neuronal cells exposed to ACM or control media, followed by MS/MS proteomic analyses. These data pointed toward a relatively small number of proteins that coimmunoprecipitated with PI3K, and surprisingly only five were regulated by the ACM signal. These hits included expected PI3K interactors, such as the platelet-derived growth factor receptor A (PDGFRA), as well as novel RNA-binding protein interactors ZC3H14 (zinc finger CCCH-type containing 14) and THOC1 (THO complex protein 1). In particular, ZC3H14 has recently emerged as an important RNA-binding protein with multiple roles in posttranscriptional regulation. In validation studies, we show that PI3K recruitment of ZC3H14 is necessary for PDGF-induced neuroprotection and that this interaction is present in primary retinal ganglion cells. Thus, we identified a novel non–cell autonomous neuroprotective signaling cascade mediated through PI3K that requires recruitment of ZC3H14 and may present a promising strategy to promote astrocyte-secreted prosurvival signals.

Astrocytes can support neuronal viability through a range of homeostatic functions, including secretions of prosurvival factors, and as mediators of metabolic and oxidative stress and tissue remodeling (1, 2, 3, 4, 5, 6). Analysis of astrocyte-conditioned media (ACM) provides a window into astrocyte secretory activities and may generate novel strategies for the treatment of neurodegenerative diseases (7, 8, 9, 10). In this context the retina provides a relatively simple and accessible system for probing this astrocyte–neuron cross talk (5, 11, 12, 13). Using a model of primary retinal astrocytes, we recently demonstrated potent neuroprotective effects of a secreted astrocyte activity (12). This activity was observed in the context of acute and chronic neuronal injury, both *in vitro* and *in vivo*, and defined methods for collecting and testing of ACM components and activity (5, 12, 13, 14). We also showed that this active ACM contains a complex mixture of protein and lipid factors (12). However, the protective neuronal mechanism integrating and transducing these diverse ACM signals is unclear. In addition, isolation and dissection of this activity remains challenging in the context of stress and injury conditions.

To address this issue, we designed a combination of functional chemical genetics screening followed by proteomics to identify key neuronal interactors mediating ACM protection in a model of neuronal injury. As a first step, we screened a kinase inhibitor library that revealed phosphoinositide 3-kinase (PI3K) as a central player transducing ACM-mediated neuroprotection from oxidative and metabolic stress. This result is consistent with evidence that homeostatic ACM contains an array of neurotrophic factors that activate class I PI3K signaling (15, 16, 17). PI3Ks are a family of lipid kinases that mediate a wide range of critical neuronal processes, focusing on bioenergetic modulation and protection against metabolic stress (18, 19, 20). Class I PI3Ks are formed as heterodimers of p85 regulatory subunits with p110 catalytic subunits and are activated by binding of p85 directly to a phosphorylated receptor tyrosine kinase, or indirectly through interactions with adapter proteins (20). Yet, the specific interactions that modulate endogenous PI3K signaling in the context of astrocyte–neuron communication are not known.

Here we utilize this untargeted approach to identify and validate a unique neuronal interactome that explores the shift in endogenous PI3K binding preferences following protective ACM exposure, compared with control cell-free media (CFM). These hits included expected PI3K interactors, such as the platelet-derived growth factor receptor A (PDGFRA), as well as the novel RNA-binding protein (RBP) interactors ZC3H14 (zinc finger CCCH-type containing 14) and THOC1 (THO complex protein 1). In particular, ZC3H14 has emerged as an important RBP with multiple roles in posttranscriptional regulation, including mRNA stability and transport (21, 22, 23, 24, 25), through interactions with THOC proteins (26). These analyses provide new insight into the role of induced neuronal and retinal PI3K through identification of novel functional interactions.

## Results

### ACM neuroprotection is mediated through PI3K signaling

**Figure 1:**
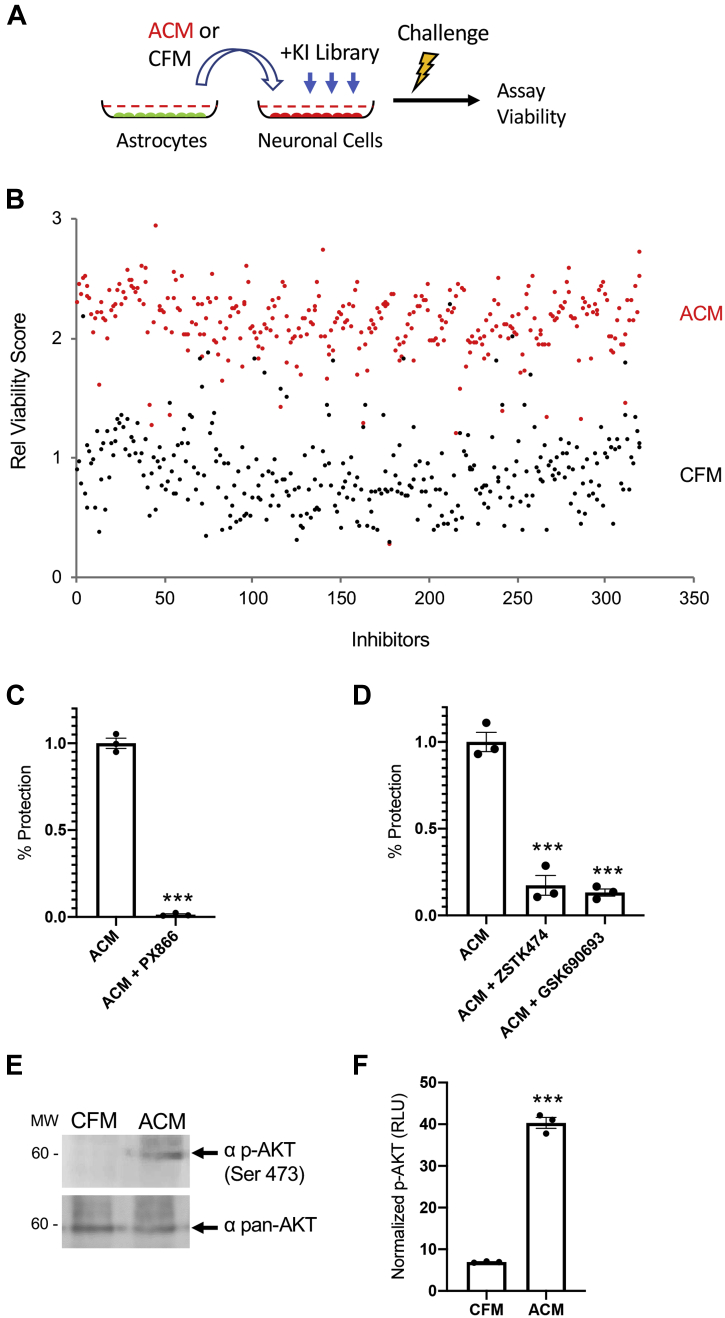
**Phosphoinositide 3-kinase** (**PI3K) is required for astrocyte conditioned media (ACM)–mediated neuroprotection.***A*, overview of screen showing collection of ACM and application to neuronal cells that are then challenged in the presence of a kinase inhibitor (KI) library and analyzed to determine their effect on cell survival. *B*, scatter plot comparing viability results for KI screened under ACM (*red*) compared with cell-free media (CFM) (*black*) conditions challenged with 5 mM glutamate. Note the separation between treatment conditions for most compounds, indicating the general window of ACM protection. *C*, validation with the PI3K inhibitor PX866 (0.25 μM), which blocked ACM-induced protection in Ht22 cells (n = 3, ∗∗∗*p* < 0.0001, bars are SE). *D*, similarly, the PI3K inhibitor ZSTK474 (1.0 μM) and AKT inhibitor GSK690693 (1.0 μM) each effectively blocks the ACM protective activity in murine primary cortical neurons (n = 3, ∗∗∗*p* < 0.0001, ANOVA F = 106.2 and *p* < 0.0001, bars are SE). *E* ACM treatment induces rapid phosphorylation of AKT compared with total AKT (pan-AKT) by 0.5 h. *F*, quantification of the results from (*E*) (n = 3, ∗∗∗*p* < 0.0001, bars are SE).

In order to identify kinase signaling that is necessary to transduce ACM-mediated neuroprotective activity, we designed a functional screen using a library of defined kinase inhibitors applied to a model of ACM-induced neuroprotection on Ht22 cells that we have optimized and reported previously (12). ACM, or control CFM, was collected from retinal astrocytes according to our established methods (5, 12, 14) and applied to neuronal Ht22 cells in the presence of each inhibitor using a robotics platform. Cells were then subjected to neurotoxic glutamate-induced oxidative challenge and the resulting effects on cell viability assessed (Fig. 1*A*). This protection is illustrated by the total screen results that show a clear distinction in viability between ACM- and CFM-treated samples, demonstrating that most of the kinase inhibitors had no effect on ACM-induced protection at 1 μM (Fig. 1*B*). It is remarkable that, of the total library, only 10 kinase inhibitors met the selection criteria showing loss of ACM-induced protection, but no apparent toxicity, as defined in the methods. Further, seven of these ten compound hits targeted the PI3K/AKT pathway, strongly implicating a role for this signaling pathway in transducing the protective ACM effect (Table 1).

**Table 1:** Kinase inhibitor hits reducing ACM protection in Ht22 cells at 1 μM are dominated by PI3K/AKT

Compound	Pathway	% Reduction in ACM Protection
None	—	0
1. Alvocidib	CDKs 1,2, 4, 6	97.7
**2. PIK-75**	**PI3K (p110α)**	**87.8**
**3. A-443654**	**Akt**	**87.4**
4. MK-1775	Wee1	81.0
**5. NVP-BEZ235**	**PI3K/mTOR**	**43.8**
**6. PX866**	**PI3K/P10**	**24.7**
**7. GSK-1904529A**	**IGF1R/IR**	**19.7**
8. KN-62	CaMk II, P2X_7_	18.6
**9. FPA 124**	**PI3K/Akt**	**15.8**
**10. ZSTK474**	**PI3K**	**12.7**

PI3K/AKT inhibitors are in bold.

ACM, astrocyte conditioned media; PI3K, phosphoinositide 3-kinase.

To validate the screening results, the effect of PI3K/AKT inhibition was verified independently using fresh compound by hand in both Ht22 cells and in primary cortical neurons. A known PI3K/AKT inhibitor, PX866 (IC_50_ = 0.1–88 nM [27]), which is a potent open ring analog of Wortmannin (28), nearly completely blocked ACM-induced neuroprotection in Ht22 cells (Fig. 1*C*). This effect is notably more robust than the original screening result (Table 1) and may reflect inconsistencies in the original compound library, automatic pipetting, or plate effects. We also reproduced the ACM neuroprotective effect in primary cortical neurons and verified that the PI3K inhibitor, ZSTK474 (IC_50_ = 0.7–210 nM) (29, 30) and the AKT inhibitor GSK690693 (IC_50_ = 20–890 nM) (31) similarly significantly reduced >80% of the neuroprotective effect (Fig. 1*D*). None of the validated kinase inhibitors was toxic when tested at the specified concentrations in either model employed. Finally, we assessed whether ACM treatment is sufficient to activate the PI3K pathway by immunoblotting for downstream AKT phosphorylation following ACM addition in Ht22 cells. ACM treatment rapidly induced AKT phosphorylation, as reflected by the significantly increased phospho-AKT signal by 30 min, normalized to total AKT (Fig. 1*E*). Taken together, these data indicate that PI3K signaling was the only robustly supported hit from the kinase inhibitor screen.

### PI3K immunoprecipitation

**Figure 2:**
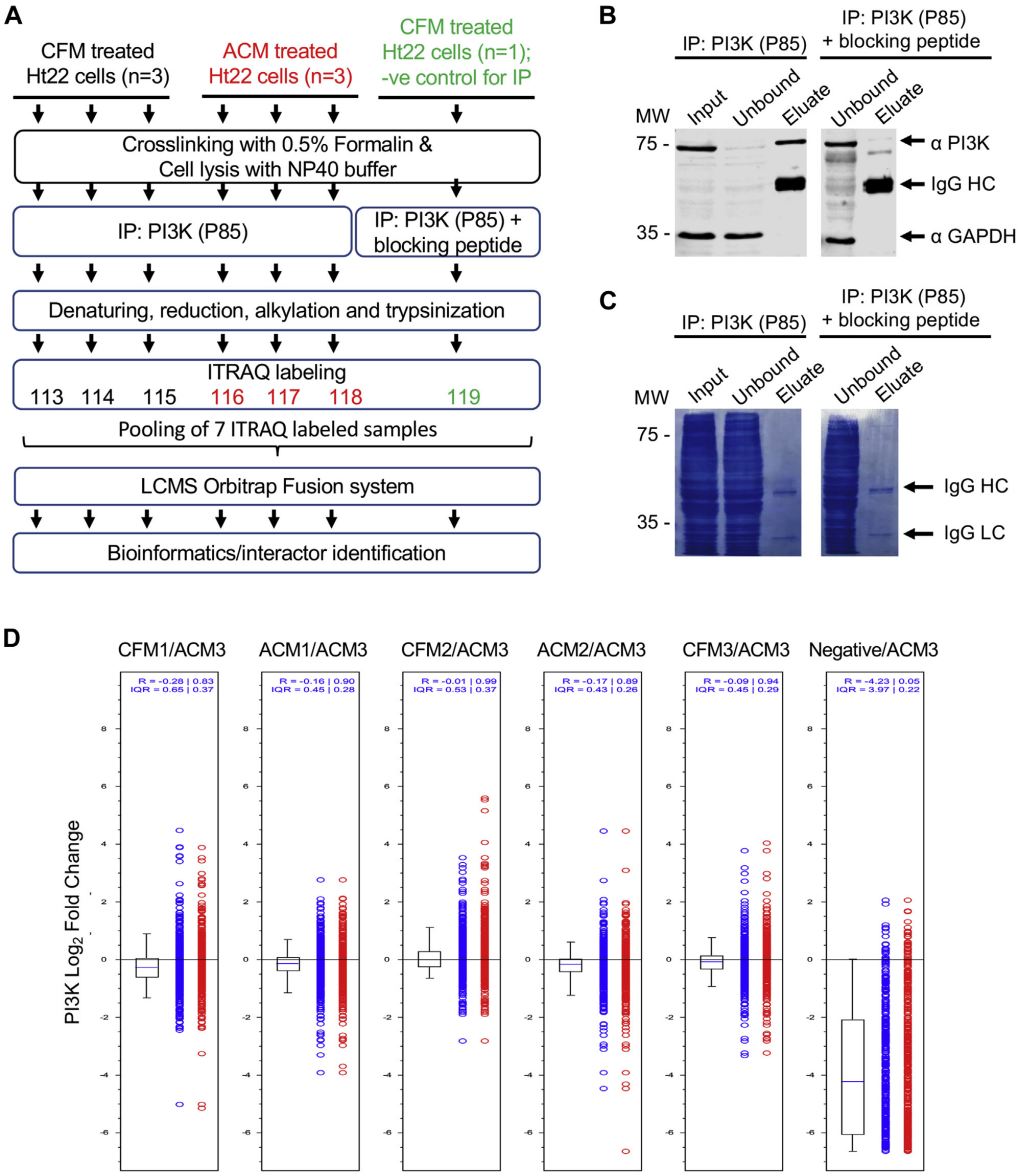
**Specific capture and analyses of neuronal phosphoinositide 3-kinase (PI3K) to determine astrocyte conditioned media (ACM)–induced interactors.***A*, a schematic of the interactome experimental design: Cell lysates were isolated from Ht22 cells treated with ACM (n = 3) or control cell-free media (CFM) (n = 3). Lysates were lightly fixed and submitted to immunoprecipitation (IP) of endogenous PI3K, along with an additional negative control with a PI3K-blocking peptide. Covalent modifications of primary amines in all seven samples were done with iTRAQ labeling, after which samples were pooled to be analyzed concurrently by MS/MS. *B*, following a PI3K antibody IP screen, Ab2 depleted PI3K in the unbound fraction and produced a strong band in the eluted fraction (*arrow*). An Ab2-blocking peptide inhibited the capture of PI3K as reflected by no depletion in the unbound fraction and a reduced PI3K band in the eluted fraction (both panels from the same blot). *C*, a corresponding Coomassie stain of the blot shows a relatively clear eluate lane compared with the input and unbound lanes, suggesting that the PI3K capture was relatively specific. *D*, box plot depicting iTRAQ quantification of target PI3K (P85) regulatory subunit relative to ACM3 in all P85 immunoprecipitates. The computed median peptide ratios and interquartile ranges are shown above the graph. P85 was abundant in all the ACM and CFM conditions, but very low in the negative control, as expected, confirming the specificity of affinity capture and negative control performance. *Blue circles* are iTRAQ ratios of individual peptide-to-spectrum matches used to determine the median ratio for the protein. *Red circles* are iTRAQ ratios of individual peptide-to-spectrum matches excluded from calculation of median ratio of the protein owing to redundancy or low signal to noise.

The strong evidence for PI3K/AKT in ACM-mediated activity, although interesting, provided limited detail into the protective mechanism, as this cascade can initiate pleotropic signaling through a variety of pathways (32, 33). Identifying the PI3K protein interactions influenced by the ACM signal would provide insight into this distinct functional outcome. Surprisingly, we discovered that endogenous PI3K interactomes had not been previously well described in neuronal cells. Therefore, in order to begin unraveling this signaling cascade we initiated a study to generate an interactome of the ACM-induced PI3K signaling complex compared with the control CFM. Our strategy was to immunoprecipitate endogenous PI3K from neuronal cell lysates, following treatment with ACM or CFM and analyze coprecipitates using mass spectrometry (Fig. 2*A*).

A panel of PI3K antibodies was first probed for their ability to effectively deplete and elute PI3K from cross-linked Ht22 cell lysates (Fig. S2 and Table S1). Based on this analysis, Ab2 (CST4257), which targets the p85 subunit of PI3K, was chosen, as it produced the strongest PI3K immunodepletion in the unbound fraction, combined with the strongest elution (Fig. 2*B* and Fig. S2). In order to differentiate true PI3K interactors from nonspecific binders, a negative control was performed by carrying out a parallel immunoprecipitation (IP) that preincubated Ab2 with its blocking peptide. Under this control condition there was minimal depletion of PI3K in the unbound fraction and a clearly reduced PI3K elution band (Fig. 2*B*). Subsequent Coomassie staining of blots showed a specific capture of PI3K, as reflected by generally clear eluate lanes compared with the corresponding input and unbound lanes (Fig. 2*C*).

Proteome Discoverer sequencing of the LC-MS data from PI3K (P85) immunoprecipitates identified 293 proteins with 12,117 peptide-to-spectrum matches (PSMs) at or exceeding 95% confidence. An inclusion criterion of at least two spectral counts was established to identify quantified proteins. Additional bioinformatic analyses further narrowed down the list of interactors to filter out nonspecific binders using the negative control as a reference and including only reproducible hits across the replicates. The initial protein list was sorted based on abundance, determined by PSM. Two key observations in these data highlight the selectivity of the affinity capture: (1) Both the target P85 regulatory and P110 catalytic PI3K subunits were the most abundant in the interactome, with more than 2000 PSMs for each hit (Table 1), indicating a highly specific IP of PI3K; and (2) the abundance of PI3K P85 subunits in the negative peptide control was at least sixfold lower than in the other experimental conditions (Fig. 2*D*). Together, these results indicate that the PI3K complex was highly purified.

### ACM-induced interactome identification

**Figure 3:**
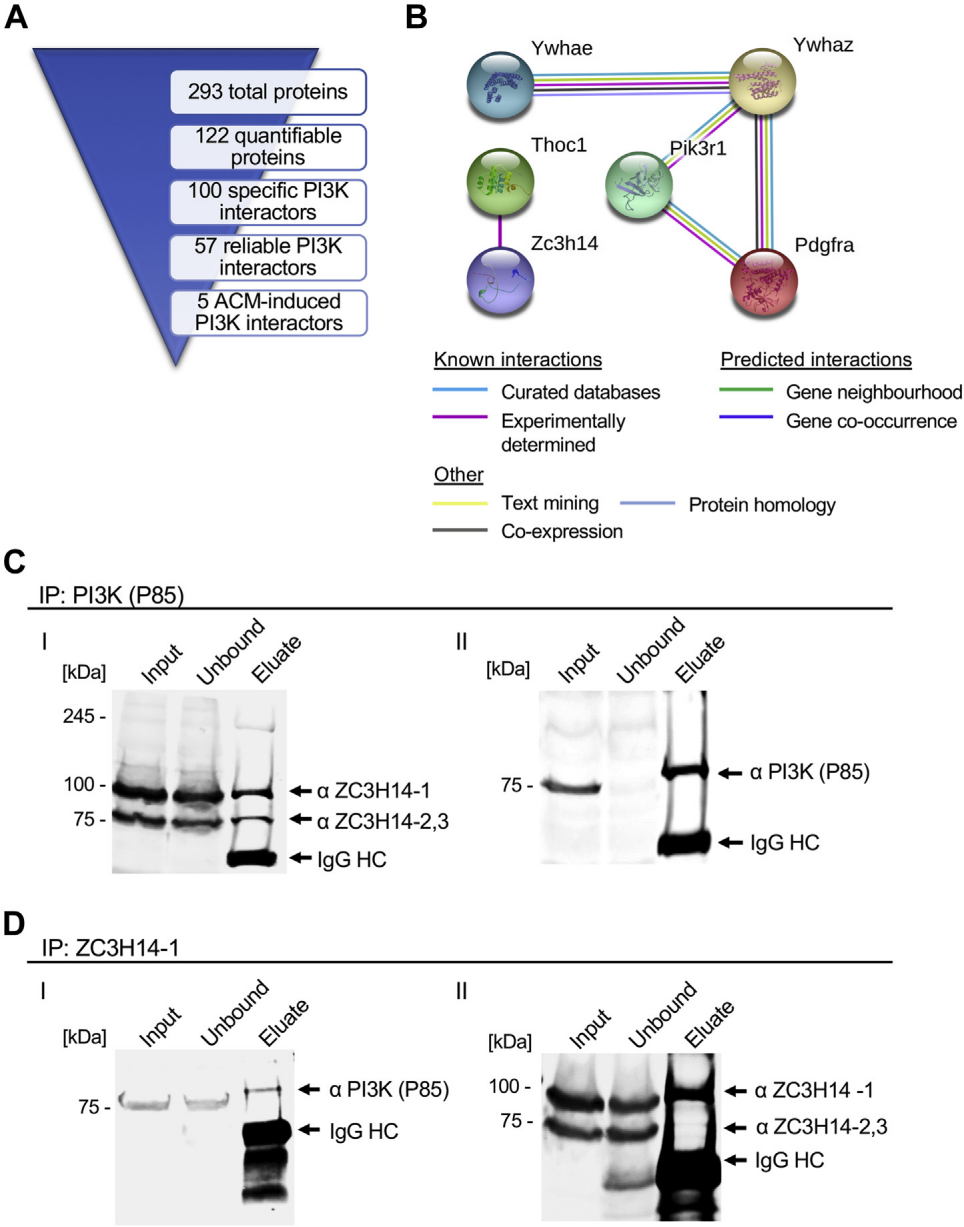
**Identification of ZC3H14 as a novel phosphoinositide 3-kinase (PI3K) interactor.***A*, overview of PI3K MS/MS hits and subsequent bioinformatic refinement of interactor lists. *B*, following analyses, a total of only five PI3K protein interactions were increased following astrocyte conditioned media (ACM) treatment. STRING analyses of the corresponding gene names revealed previously established interactors of PI3K P85 (Pik3r1), such as the receptor tyrosine kinase PDGFRA (Pdgfra), and 14-3-3 adaptor proteins Ywhaz and Ywhae through a variety of indicated sources. In addition, two novel ACM-induced interactors were detected; the poly(A) RNA-binding proteins (RBPs) ZC3H14 (Zc3h14) and THOC1 (Thoc1). *C*, validation of ZC3H14 binding was performed by coimmunoprecipitation of PI3K from Ht22 cell lysates and probed with an antibody to ZC3H14 (*I*). Successful capture of PI3K was confirmed by blotting for PI3K (*II*). *D*, as a further validation for the interaction a reverse coimmunoprecipitation was carried out by capturing ZC3H14-1 and probing for PI3K (*I*). Successful ZC3H14-1 capture was confirmed by immunoblotting for ZC3H14 isoforms (II).

To facilitate further analyses, we designed a progressive refinement of the interactor list (Fig. 3*A*). The preliminary results yielded a total of 122 proteins reliably quantified from three or more MS3 spectra with reporter ion signals representing all six immunoprecipitated groups (all CFM- and ACM-treated replicates) (see full list in Table S3). Of these, 100 proteins had negative control/ACM3 iTRAQ ratios of less than 0.40, indicating that they were specifically co-enriched with PI3K P85 (gene name PI3KR1). Furthermore, 57 candidate PI3KR1 interactors fit reproducibility criteria of median reporter ion signals varying less than 35% among all three ACM replicates. This list represents the top interactors with high confidence across all samples (Table 2; top 20 hits shown). Finally, from this group we investigated how PI3K binding preferences shifted following ACM treatment, by identifying interactors with a CFM1/ACM3, CFM2/ACM3, and CFM3/ACM3 ratio less than 0.65 or higher than 1.35, indicating that ACM regulated binding with PI3K. Remarkably, a total of only five interactors met all of these criteria, each showing increased binding to PI3K following ACM treatment compared with CFM treatment (Table 3, Fig. S3).

**Table 2:** Top 20 PI3K interactome hits, sorted by spectral counts in six samples

Accession	Description	PSM	CFM1/ACM3	ACM1/ACM3	CFM2/ACM3	ACM2/ACM3	CFM3/ACM3	Negative ctrl/ACM3
**IPI00263878.2**	**Phosphatidylinositol 3-kinase (PI3K ) regulatory subunit alpha**	**2821**	**0.826**	**0.896**	**0.995**	**0.891**	**0.938**	**0.106**
**IPI00136110.4**	**Phosphatidylinositol-4,5-bisphosphate 3-kinase catalytic subunit beta isoform**	**2108**	**0.83**	**0.885**	**1.078**	**0.804**	**0.926**	**0.114**
IPI00331708.3	Isoform 1 of MKL/myocardin-like protein 1	1551	0.9	0.858	1.074	0.906	1.016	0.069
IPI00323357.3	Heat shock cognate 71-kDa protein	735	0.99	0.853	1.126	0.822	0.904	0.133
IPI00119627.1	Insulin receptor substrate 1	488	0.69	0.866	0.846	0.861	0.734	0.124
IPI00379844.5	Insulin receptor substrate 2	359	0.753	0.885	0.934	0.851	0.744	0.278
IPI00117159.2	Phosphatidylinositol 3-kinase regulatory subunit beta	171	0.681	0.84	0.925	0.98	0.926	0.059
IPI00230632.2	Isoform Cas-A of breast cancer anti-estrogen resistance protein 1	148	1.152	0.954	1.204	0.964	1.04	0.289
IPI00406794.2	GRB2-associated binding protein 1	106	0.718	0.847	0.785	0.892	0.603	0.097
IPI00319992.1	78-kDa glucose-regulated protein	102	0.858	0.804	1.064	0.806	0.788	0.075
IPI00554929.3	Heat shock protein HSP 90-beta	91	0.865	0.843	0.942	0.868	0.733	0.18
IPI00223902.2	Isoform 2 of uncharacterized protein KIAA1310	66	0.84	1.052	0.965	1.139	0.874	0.013
IPI00987441.1	Uncharacterized protein (fragment)	60	1.497	1.028	1.233	1.085	1.724	0.292
IPI00139780.1	60S ribosomal protein L23	42	0.696	0.832	0.883	0.866	0.788	0.072
IPI00323806.4	Putative uncharacterized protein	33	0.935	0.937	1.156	0.865	0.94	0.23
IPI00653962.1	Uncharacterized protein	30	1.246	0.906	1.809	0.816	1.94	0.089
IPI00762542.2	40S ribosomal protein S11	29	0.897	0.917	0.881	0.842	0.753	0.108
IPI00465880.4	40S ribosomal protein S17	27	0.706	0.922	0.963	0.949	0.558	0.164
IPI00466258.2	Isoform 1 of SH3 domain–containing kinase-binding protein 1	25	0.786	0.989	1.009	0.934	0.556	0.126
IPI00986371.1	60S ribosomal protein L27a-like	20	1.679	0.8	1.379	0.83	0.846	0.258

The ratio columns represent average iTRAQ intensity ratios observed for a given protein. Hits were filtered to include proteins with an ACM1/ACM3 and ACM2/ACM3 ratio between 0.65 and 1.35, to ensure high reliability, and a 0.4 negative control/ACM ratio to eliminate nonspecific binders. Note that the most abundant hits are the regulatory (P85) and catalytic (P110) PI3K subunits.

Bold items indicate the primary PI3K subunit capture targets.

**Table 3:** List of ACM-induced PI3K interactors

Accession	Description	PSM	Sum of peptides	CFM1/ACM3	CFM2/ACM3	CFM3/ACM3	Average fold change	Negative/ACM
IPI00461416.4	Isoform 1 of zinc finger CCCH domain–containing protein 14 (**Zc3h14**)	29	99	0.48	0.56	0.54	**0.53**	0.186
IPI00844650.1	Isoform 1 of alpha-type platelet-derived growth factor receptor (**Pdgfra**)	24	70	0.62	0.52	0.45	**0.53**	0.392
IPI00114407.2	Isoform 1 of THO complex subunit 4 (**Thoc1**)	15	60	0.31	0.44	0.38	**0.38**	0.03
IPI00116498.1	14-3-3 protein zeta/delta (**Ywhaz**)	7	52	0.42	0.51	0.42	**0.45**	0.129
IPI00118384.1	14-3-3 protein epsilon (**Ywhae**)	6	41	0.42	0.51	0.43	**0.45**	0.143

In addition to meeting the criteria outlined in Table 2, these interactors had a CFM1/ACM3, CFM2/ACM3, and CFM3/ACM3 ratio between 0.65 and 1.35, indicating ACM regulated binding with PI3K.

Bold indicates the corresponding gene names for each interactor.

ACM, astrocyte conditioned media; PI3K, phosphoinositide 3-kinase.

Of these five ACM interactors, STRING analyses of the corresponding gene names intriguingly revealed both previously established and novel interactors (Fig. 3*B*). Previously identified interactors included two 14-3-3 adaptor proteins, which are critical to propagating PI3K pathway signaling by stabilizing the insulin receptor substrate-2 (IRS2) (34, 35, 36). In addition, PDGFRA has been recently shown to promote potent retinal and neuronal prosurvival effects (37, 38, 39, 40, 41), providing an intriguing potential connection between ACM extracellular signals and PI3K activation (42, 43). However, in many ways the most interesting result was the identification of two previously unknown PI3K interactors induced by ACM, isoform 1 of zinc finger CCCH domain-containing protein 14 (ZC3H14-1) and isoform 1 of THO complex subunit 4 (THOC1). Intriguingly, both of these are RBPs with recently described interactions between them to coordinate mRNA processing and stabilization in neuronal cells (24, 25, 26).

### ZC3H14 complexes with PI3K

In the fully processed data set, ZC3H14 was the most highly regulated ACM-induced interactor, and to our knowledge, this protein has not been previously investigated in the context of PI3K signaling. ZC3H14 modulates several steps in the posttranscriptional regulation of gene expression, including regulating polyadenosine (poly(A)) tail length and nuclear export (22, 24, 26). Therefore, a PI3K–ZC3H14 interaction could be consistent with the roles of PI3K/AKT signaling in promoting cell survival via increased protein synthesis (15, 44). To confirm the interaction between PI3K and ZC3H14 biochemically, coimmunoprecipitations were carried out by capturing PI3K and probing for ZC3H14 (Fig. 3*C*). Effective PI3K capture and elution was verified by probing a parallel blot (Fig. 3*C*). ZC3H14 is alternatively spliced to produce several protein isoforms. The largest isoform, described as ZC3H14 isoform 1, was identified in the interactome, but it may also encompass isoforms 2 and 3 (22, 45). This experiment revealed that ZC3H14 isoforms 1 and 2/3 coimmunoprecipitated with PI3K. In order to confirm the identity of the eluted band, ZC3H14-1 was knocked down using a previously validated siRNA (46) (Fig. S4). Finally, as further confirmation, a reverse coimmunoprecipitation of the two proteins was carried out by capturing ZC3H14, followed by probing for PI3K p85, which further supported the interaction (Fig. 3*D*). In this case a control blot verified ZC3H14 capture and elution (Fig. 3*D*). These data provide further biochemical evidence to support an interaction between ZC3H14 and PI3K.

### PDGF induces neuroprotective PI3K recruitment of ZC3H14

**Figure 4:**
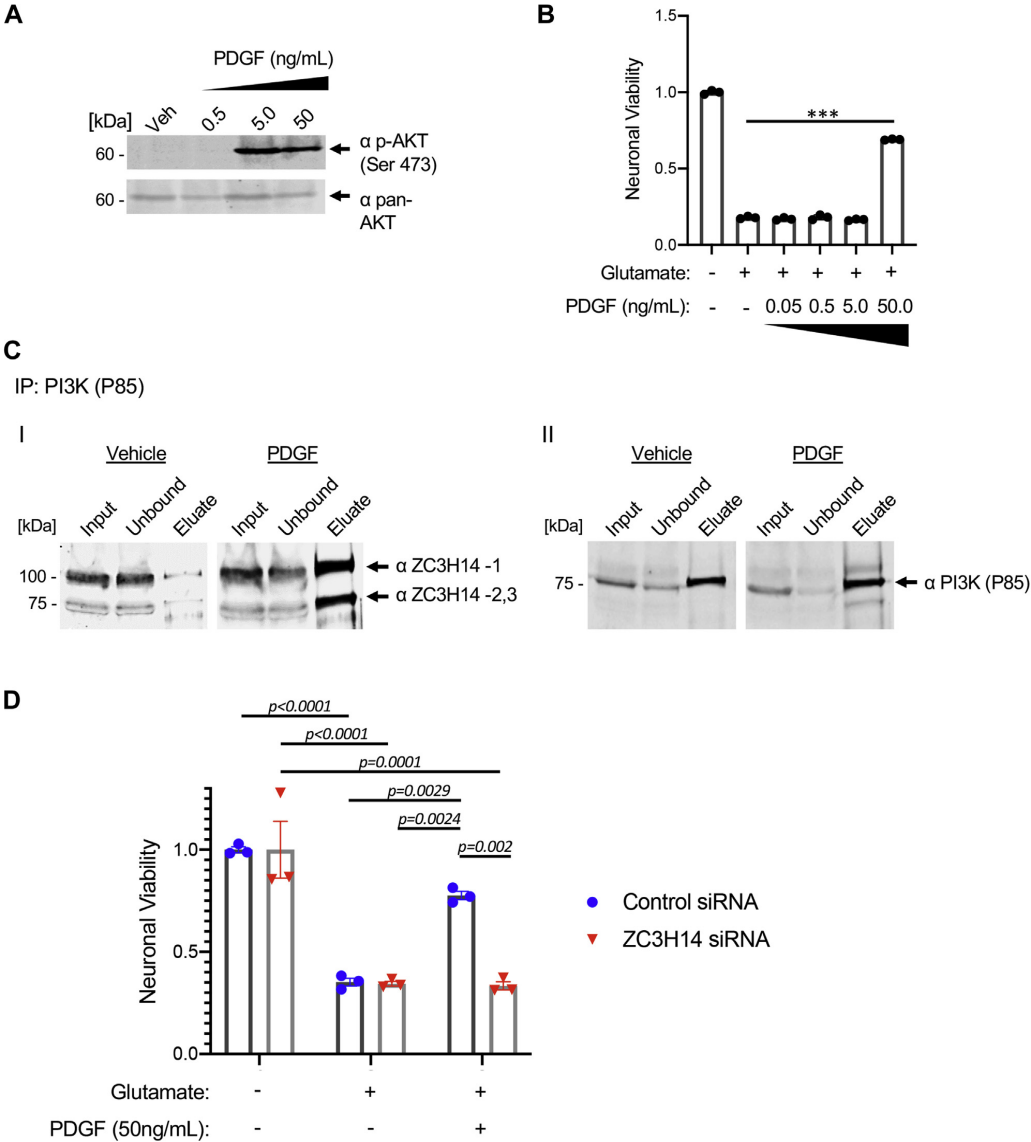
**Phosphoinositide 3-kinase (PI3K) recruitment of ZC3H14 is necessary for platelet-derived growth factor (PDGF)–induced neuroprotection.***A*, recombinant PDGFbb treatment produces a strong p-AKT signal in Ht22 cells at 5 and 50 ng/ml after 30 min. *B*, a robust cytoprotective effect is mediated by recombinant PDGFbb at 50 ng/ml against glutamate injury (n = 3, ∗∗∗*p* < 0.0001, ANOVA F = 4021 and *p* < 0.0001, bars are SE). *C*, PDGFbb treatment of neuronal cells induces increased coimmunoprecipitation of ZC3H14 isoforms (*arrows*) from PI3K capture (*I*). Immunoblotting of PI3K was also carried out to confirm equal amounts of captured and eluted PI3K (*arrow*) in both conditions (*II*). *D*, ZC3H14 knockdown eliminates PDGF-mediated neuroprotection against glutamate injury (n = 3, post hoc p values indicated on chart, bars are SE, two-way ANOVA for treatment F = 63.75 and *p* < 0.0001, knockdown F = 9.722, *p* = 0.0089, interaction F = 9.208 and *p* < 0.0038).

In addition to ZC3H14, the MS/MS interactome results suggested that ACM treatment increases PDGFRA binding to PI3K. These results were interesting because they provided a potential direct link between PI3K signaling and PDGF, present as one of several growth factors detected in the ACM milieu that likely contribute to an integrated ACM response (Fig. S5). In addition, the activity of PDGF isoforms has been associated with neuroprotective effects on retinal ganglion cells and cortical neurons (37, 38, 39, 40, 41, 47). This established link could therefore provide a more precise probe to further investigate downstream PI3K interactions than ACM. To validate this finding, we first investigated whether exogenous PDGF activates the PI3K pathway in HT22 cells. The previous literature has indicated that PDGFbb isoforms have potent neuroprotective activity in oxidative and metabolic injury models (48). Addition of recombinant PDGFbb rapidly induced robust AKT phosphorylation by 30 min at both 5 and 50 ng/ml, indicating activation of the PI3K pathway (Fig. 4*A*). Next, we investigated whether recombinant PDGFbb is sufficient to generate HT22 protection from the glutamate injury model previously used for screening. The experiment demonstrated a robust corresponding protective effect mediated by recombinant PDGFbb at 50 ng/ml (Fig. 4*B*). It is unclear why only the higher concentration elicited a protective effect, but the functional assay is performed at a much later time point than the phosphorylation assay, leading to potential discrepancies in phosphorylation time course and the roles of additional phosphorylation sites or complexed proteins, among other variables. Finally, we tested the addition of the receptor tyrosine kinase inhibitor Ki 8751, which targets PDGFRA (IC_50_ = 67 nM), along with VEGFR-2 (49). Ki 87512 treatment reduced the ACM-mediated protection in a dose-dependent manner (Fig. S6).

As PDGFRA and ZC3H14 were both components of the PI3K complex in the ACM interactome, we then investigated whether PDGF treatment affects PI3K–ZC3H14 binding. A PI3K coimmunoprecipitation was carried out as previously, following treatment with 20 ng/ml PDGFbb or vehicle. For each IP, equal concentrations of cell lysate, antibody, and beads were used. The resulting blots show a dramatic increase in ZC3H14 elution with treatment of PDGF (Fig. 4*C*, *I*). Of note, this observation is supported by complementary ZC3H14 depletion in the unbound fraction of PDGF-treated cells compared with the vehicle control (Fig. 4*C*, *I*). As a control, depletion and elution of PI3K itself was consistent for each condition (Fig. 4*C*, *II*).

To test whether ZC3H14 is required for the PDGF-induced protection of cells, we used a validated siRNA (46) (Fig. S4) to deplete ZC3H14 and examined cell response to glutamate injury. Although cells treated with the scrambled control siRNAs still exhibited PDGF-induced protection, loss of ZC3H14 rendered the cells unresponsive to PDGF-induced protection (Fig. 4*D*). This experiment demonstrates that ZC3H14 is critical for PDGF-induced protection. Taken together, these data suggest that PDGF treatment increases PI3K recruitment of ZC3H14 to mediate its protective signal.

### Interaction between PI3K and ZC3H14 in retinal ganglion cells

**Figure 5:**
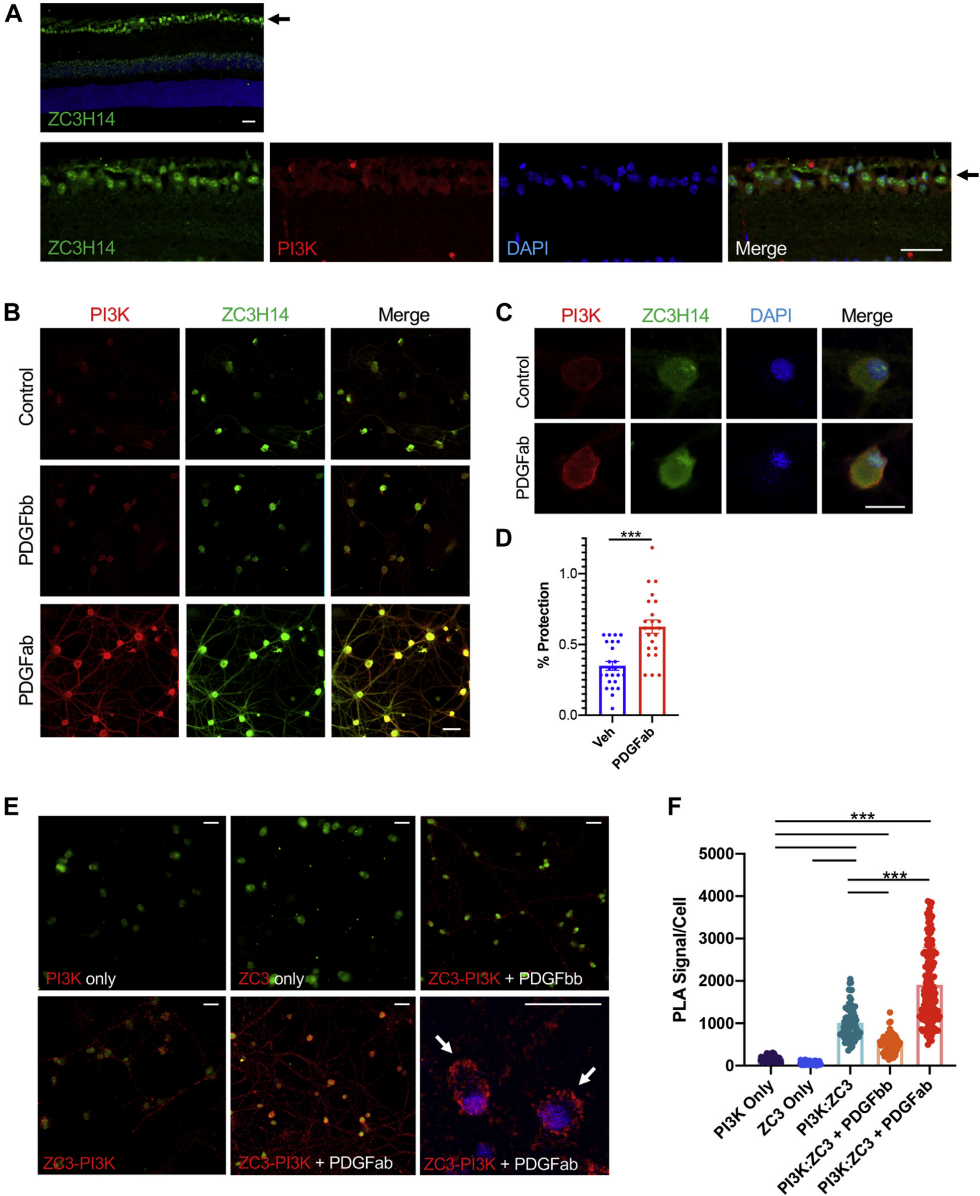
**Interaction between phosphoinositide 3-kinase (PI3K) and ZC3H14 in retinal ganglion cells.***A*, naive mouse retinal sections probed with antibodies to PI3K (*red*) and ZC3H14 (*green*) show that they partially colocalize in the retinal ganglion cell layer (*arrow*). The top panel shows a lower magnification image of the whole retina highlighting the prominent ZC3H14 signal in the ganglion cell layer (*arrow*). *B*, partial colocalization of PI3K (*red*) and ZC3H14 (*green*) in primary retinal ganglion cells. Treatment with 50 ng/ml PDGFab, but not PDGFbb, induces increased colocalization of both proteins throughout somas and neurites (*yellow merge*). *C*, higher-magnification confocal slice images through the soma and nucleus showing that the ZC3H14 staining is concentrated in the nucleus, and is also present throughout the cytoplasm, particularly upon treatment with PDGFab. *D*, PDGFab treatment (150 ng/ml) significantly protects RGCs from oxidative stress injury compared with the vehicle (veh) (∗∗∗*p* < 0.0001, bars are SE). *E*, proximity ligation assay (PLA) results show no proximity signal (*red*) when either PI3K or ZC3H14 (ZC3) antibody is used alone. Note: *green staining* in all panels is Neu-N, used as a cell marker. PLA reaction with both antibodies for (PI3K and ZC3H14) results in a *red* proximity signal throughout the soma and neurites. Treatment with PDGFab causes a dramatic increase in cytoplasmic PLA signal (high-magnification confocal slice inset compares PLA signal with *blue* DAPI staining). *F*, quantification of PLA results for each condition confirms a highly significant PLA interaction that is increased by PDGFab treatment. (∗∗∗*p* < 0.0001 for all comparisons, ANOVA F = 218.9 and *p* < 0.0001, bars are SE) (The scale bars represent 40 μm).

To assess whether these ACM-induced interactions are relevant in the context of primary neurons, we assessed PI3K and ZC3H14 colocalization in retinal ganglion cell (RGCs). The retina provides an excellent model to study this interaction given the close association between RGCs and neighboring astrocytes. Immunostaining of mouse retinal sections revealed prominent localization of PI3K and ZC3H14 to the ganglion cell layer (Fig. 5*A*), as well as colocalization between ZC3H14 and THOC1 (Fig. S7). Of note, it seemed that the ZC3H14 staining was largely nuclear with some cytoplasmic staining, whereas the PI3K staining was cytoplasmic (Fig. 5*A*). The same subcellular localization was observed in cultured RGCs under control conditions (Fig. 5*B*). This pattern is consistent with previous reports showing primarily a nuclear localization of ZC3H14 (23) and was not strongly affected by treatment with PDGFbb in RGCs. However, the PDGFab isoform has been reported to be a potent neuroprotective factor for RGCs (40, 41). Upon treatment with PDGFab there was an increase in cytoplasmic colocalization of ZC3H14 and PI3K (Fig. 5, *B* and *C*). This result reproduces previous studies that have detected ZC3H14 in the cytoplasmic fraction of neurons (24, 50). Of interest, PI3K signal was also increased by PDGFab treatment, suggesting that additional mechanisms are present that increase PI3K expression. Finally, the same PDGFab treatment was observed to be protective to RGCs challenged with an oxidative stress injury (Fig. 5*D*).

To test for direct interactions between endogenous PI3K and ZC3H14 in primary RGCs, which are more limited in number than immortalized cells, we employed a proximity ligation assay. This technique generates an amplified fluorescent signal only when the target proteins identified by two antibody probes are in direct proximity, indicating a direct interaction with high subcellular specificity (51). Application of either PI3K or ZC3H14 probes alone showed no proximity signal. However, application of both PI3K and ZC3H14 probes produces a clear proximity ligation assay (PLA) signal localized to RGC somas and neurites (Fig. 5*E*). PDGFbb treatment produced little difference in this signal. However, treatment with PDGFab resulted in a striking increase in the PLA signal, particularly in the perinuclear region (Fig. 5*E*). Quantification of these results confirmed them be highly significant and reproducible (Fig. 5*F*). Therefore, endogenous PI3K and ZC3H14 interact in RGCs, and this interaction is enhanced by PDGFab.

## Discussion

**Figure 6:**
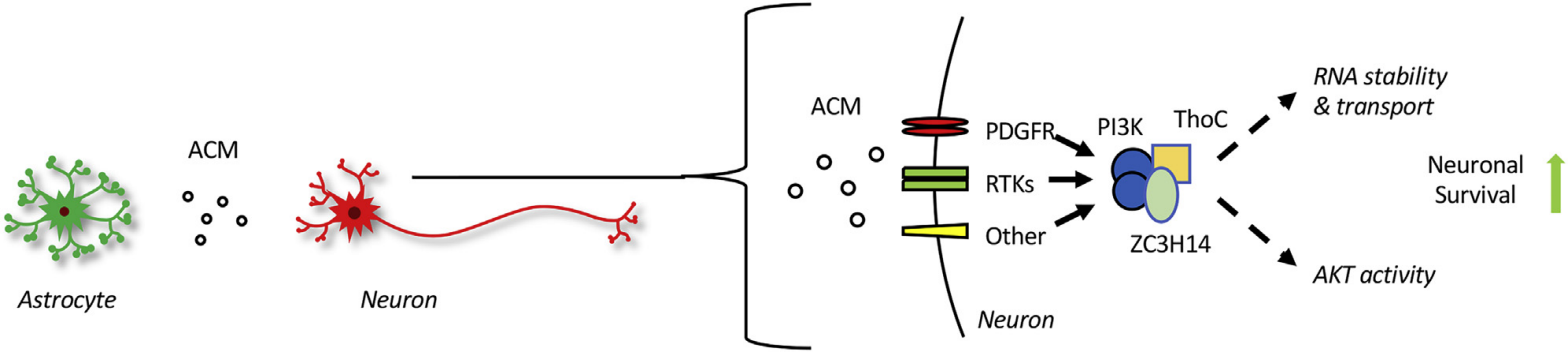
**Proposed interaction scheme in the context of astrocyte-induced neuroprotection.** The astrocyte secretome contains a mixture of growth factors and lipid mediators. Multiple receptor tyrosine kinases (RTKs), including the platelet-derived growth factor receptor (PDGFR) and other potential receptors, converge to induce PI3K signaling that initiates formation of ZC3H14 signaling complex to modulate downstream effectors and RNA stability mediating neuronal survival.

A variety of homeostatic and prosurvival astrocyte activities have recently been described, strengthening the concept of key glioprotective central nervous system mechanisms (2, 52, 53, 54, 55). In previous work, we established that the astrocyte secretome contains potent neuroprotective factors that ameliorate metabolic and excitotoxic neuronal injury in *in vitro* and *in vivo* models (12). Analysis of ACM is an established approach to investigating astrocyte–neuron interactions and identifying novel astrocyte-derived paracrine factors and signaling (12, 56, 57, 58). Yet, dissecting the complex activities present in ACM has proven challenging, and the resulting protective neuronal response pathways have remained unclear. To address this point, we used a combination of chemical genetics screening and mass spectrometry to identify key neuronal signaling pathways and ligands involved in ACM-mediated protection against metabolic stress. From a broad spectrum of targets, our screening identified PI3K as a key necessary signaling hub for ACM-induced neuroprotective activity, whereas the subsequent interactome identified only five proteins, all increased in response to ACM treatment. Encouragingly, these results included expected interactors, such as 14-3-3 adaptor proteins and PDGFRA, as well as a novel interaction with RNA-binding proteins ZC3H14 and THOC1 (Fig. 6). Therefore, this approach revealed a remarkably specific astrocyte–neuronal signaling network, considering the diversity present in the ACM milieu.

The astrocyte secretome contains a complex mixture of proteins, lipids, neurochemicals, and nucleic acids (7, 8, 12, 59). Isolation and analysis of these components can be challenging, and our own analyses of ACM fractions revealed enrichment in a broad variety of growth factors, including bFGF, NGF, PDGF, CNTF, BDNF, and lipid mediators (Fig. S5 and (4, 12), all with potential neuroprotective activities. The identification of PDGFRA in our interactome provided an interesting connection, consistent with these extracellular cues and PI3K signaling, and suggested the use of PDGF as stimulus for further validation studies. PDGF isoforms are dimers of a- and b-chains (aa, bb, or ab), which signal with variable affinities through tyrosine kinase receptors, including PDGFRA (60, 61). In a neuronal context, PDGF isoforms are secreted by macroglia, including astrocytes and retinal Müller cells, as well as by neurons, mediating short-range paracrine communication (62). PDGF, in addition to having well-established vascular functions, has more recently been demonstrated to protect against oxidative and metabolic stress in primary neurons, including the potent activity of PDGFab on retinal ganglion cells and hippocampal neurons (37, 38, 39, 40, 41). We demonstrated the ability of PDGF isoforms to induce PI3K activation and generate a ZC3H14-dependent neuroprotective effect. However, the diverse factors present in ACM suggest that *in situ* multiple secreted signals could be integrated through diverse receptors to activate PI3K and initiate this neuroprotective cascade (Fig. 6).

Activation of the PI3K pathway with PDGF and other neurotrophic factors can promote cell survival through inhibition of proapoptotic signaling (19, 63), modulation of metabolic regulators (64, 65), and activation of mammalian target of rapamycin (63, 66). Less well known are protective PI3K interactions with a range of RBPs to stabilize transcripts via the adapter protein 14-3-3 (67, 68). RBPs play an important role in posttranscriptional gene regulation through physical interactions with target RNA transcripts (69, 70), among other functions, ultimately altering transcript processing, localization, and/or stability (71, 72, 73). Thus, changes in the localization or activity of RBPs can modulate gene expression in response to extracellular stimuli (44). Previously, several RBPs, including KSRP, BRF1 (or TIS11b), NF90, CELF1, and YB1, have been defined as targets of AKT-dependent signaling pathways (44). Thus, one possibility is that a signaling cascade alters the localization, function, or RNA-binding capacity of the ZC3H14 RBP to allow cells to mount a response to injury. Dysfunction of RBP activity has been increasingly observed in the context of neurodegenerative disease (74, 75, 76), and mutations in genes encoding RBPS are associated with amyotrophic lateral sclerosis (77), epilepsy (78), and Parkinson's disease (79), among others. However, this mechanism has not been previously directly linked to poly(A) RNA-binding proteins through PI3K and has not been studied in the context of astrocyte–neuron interactions.

ZC3H14 binds with high affinity to poly(A) RNA and contributes to a range of posttranscriptional functions (21, 80), including control of poly(A) tail length (22), nuclear export (81), and mRNA splicing (82). In addition, we recently reported that ZC3H14 coordinates these activities with subunits of the RNA processing THO complex, including THOC1 (26). Of interest, mutation of human and mouse ZC3H14 are associated with neurological and cognitive dysfunction (23, 24). Similarly, mutations in genes encoding THOC components are also associated with brain disorders (83, 84, 85). However, these RBPs have not been previously linked to neurodegeneration or non–cell autonomous astrocyte–neuron interactions. In our untargeted neuronal mass spectrometry data both ZC3H14 and THOC1 were identified as novel PI3K interactors induced by ACM exposure. As ZC3H14 was the stronger interactor, our subsequent experiments further validated this observation in response to neuroprotective ACM, and in primary RGC neurons. Importantly, we show that ZC3H14 is required to confer the PDGF-induced protection against glutamate injury. This result shows that ZC3H14 is functionally important in this pathway. Thus, these results provide new insight into the role of PI3K and ZC3H14 in regulating neuronal survival. The cytoplasmic and perinuclear localization for these interactions in our PLA data is intriguing and consistent with proposed roles for ZC3H14 in the cytoplasm of neurons (24, 50).

Taken together, these findings suggest a model in which diverse astrocyte cues can be integrated to alter neuronal function by regulating ZC3H14/THOC activity (Fig. 6). However, several challenging new questions are also raised, such as which downstream neuronal transcripts are altered to influence neuronal survival through the PI3K-ZC3H14 interaction? Also, the impact of altered astrocyte activation state and secretome on this mechanism is unclear. We previously identified mRNA transcripts regulated by ZC3H14–THOC binding in which *dysfunction* is linked to neurodegenerative disease, including postsynaptic density protein 95 (*Psd95*), ATP synthase lipid-binding protein (*Atp5g1*), and microtubule-associated protein tau (*MAPT*) (26). Furthermore, the 14-3-3 adapter molecules that emerged as ACM-regulated PI3K interactors serve as a compelling follow-up target, given their recently discovered role in promoting neurite regeneration in the optic nerve (86). Given the complex etiology of neuronal dysfunction, this novel interactome may reveal unconventional opportunities for therapeutic modulation of these prosurvival signals.

## Experimental procedures

### Cell cultures and treatments

All animal care and experimental procedures were approved by the University Health Network animal care committee and were in compliance with the Association for Research in Vision and Ophthalmology Statement for use of animals in vision research. Retinal astrocytes were isolated and cultured as previously described (11, 14). Briefly, retinas were dissected from adult Wistar rat eyes and placed in ice-cold Eagle's Minimal Essential Medium (Wisent) supplemented with 10% fetal bovine serum (FBS)/1% penicillin/streptomycin. Retinas were dissociated with a papain dissociation system (Worthington), after which they were triturated, counted, and seeded in astrocyte growth media (Lonza) on the first day. The medium was changed the following day to Eagle's Minimal Essential Medium supplemented with 5% FBS, 5% horse serum, and 1% penicillin/streptomycin. At 80% confluency, cells were placed on a rotating shaker for 6 to 8 h to remove microglia and then replated in 6-well plates at 1.5 × 10^5^ cells/well. ACM or cell-free control medium (CFM) was harvested after 24 h incubation and stored at –80 °C. Neuronal Ht22 cells were cultured in high-glucose Dulbecco's modified Eagle's medium (DMEM) (Sigma) supplemented with 10% FBS/1% penicillin/streptomycin (87). Primary cortical neurons were cultured as previously described (88). Briefly, cortices from E16 to 18 mouse embryos were isolated and cleaned in Hank's balanced salt solution on ice. The tissue was mechanically homogenized and dissociated with a papain dissociation system (Worthington). Neurons were maintained in Neurobasal-A media with l-glutamine and B27 supplement without antioxidants (Thermo). Primary RGCs were isolated and cultured from p7-9 Wistar rats as described (5, 12), using a magnetic MicroBead Isolator Kit according to the manufacturer's protocol (Miltenyi Biotec). To induce PI3K activity with recombinant murine PDGF-BB, Ht22 cells were plated in 10-cm dishes in reduced 5% FBS DMEM to minimize background phosphorylation (89). On the following day, Recombinant Murine PDGF-BB (Peprotech, 315-18) was added to cells in serum-free media at three different concentrations: 0.5, 5, 50 ng/ml. One hour later, cell lysates were collected in NP-40 lysis buffer, containing 150 mM Tris, 150 mM NaCl, in addition to protease/phosphatase inhibitor cocktail. Kinase inhibitors were added to cell media at over a range of concentrations, using IC_50_ values as the guideline. Primary RGCs were treated with 150 ng/ml PDGFab or vehicle 2 h prior to oxidative stress induced by 30 μM Paraquat. After 24 h the cells were fixed in 4% paraformaldehyde and processed for immunofluorescence. The numbers of surviving RGCs were determined by counting tubulin-βIII-positive cells using confocal microscopy.

### Kinase inhibitor library screen

A tool library of kinase inhibitors was used in a primary chemical screen performed at the SMART Robotics Facility at the Lunenfeld-Tanenbaum Research Institute. Ht22 cells were seeded in duplicate 384-well plates at 3000 cells per well. One plate was treated with ACM, and a corresponding plate treated with control CFM collected under identical conditions. A dilution curve established that a significant and reproducible protective activity was still measurable under these conditions (Fig. S1). A 480 tool compound kinase inhibitor library (BIOMOL) was applied to each set of plates at 1 μM along with vehicle control, followed by challenge with 5 mM glutamate, which on Ht22 cells has been used extensively as a model of glutamate-induced oxidative injury (90, 91, 92). Cell viability was assessed 20 h later using an XTT Assay Kit (Roche) and absorption read at 450 nM. Wells with media only were analyzed for background correction. Ranking of compound hits was based on two criteria: (1) The ability of each compound to reduce ACM-induced neuroprotection, observed at 102.8%, and (2) for the same compound, a <10% change on survival in CFM-treated control wells, indicating no inherent toxic effect. Validation of PI3K as a key target was performed by carrying out the same glutamate challenge assay and ACM treatment with Ht22 cells or primary neurons. In this case, cells were seeded at 5000 cells per well in 96-well plates followed by treatment with the indicated concentrations of inhibitor or dimethyl sulfoxide vehicle followed by 5 mM glutamate challenge and XTT readout for cell viability after 20 h of incubation.

### Immunoblotting

Cell lysates for Western blots were collected in either standard radio-immunoprecipitation assay buffer buffer for denaturing applications or NP-40 (described under immunoprecipitations below) for nondenaturing applications, supplemented with protease/phosphatase inhibitor cocktail. Protein concentrations were quantified using bicinchoninic acid assay (Thermo), and equal amounts were loaded for SDS-PAGE. Proteins were transferred to polyvinylidene fluoride membrane and blocked with 5% bovine serum albumin in Tris-buffered saline with 0.1% Tween 20 and incubated overnight at 4  °C with primary antibodies raised against PI3 Kinase p85 (CST), pan AKT (CST), Phospho-Akt (Ser473) (CST), GAPDH (Calbiochem), ZC3H14 (45), and THOC1 (Abcam). Membranes were then washed three times with TBS-t and probed with appropriate IRDye secondary antibody (Li-Cor Biosciences). Blots were imaged and analyzed with an Odyssey infrared imaging system (Li-Cor Biosciences), with each band being normalized to internal control.

### Immunoprecipitations

To optimize PI3K capture, Ht22 cells were plated in 15-cm dishes and grown to 70% confluency. Following a 1-h incubation with either ACM or CFM, cells were fixed with 0.5% formalin in PBS for 10 min to preserve labile and transient protein–protein interactions. Formalin was neutralized with 150 mM Tris base, 150 mM glycine solution for 10 min. Cell lysates were collected in 0.2% NP-40 lysis buffer, containing 150 mM Tris, 150 mM NaCl, with addition of protease/phosphatase inhibitor cocktail. A library of PI3K antibodies (CST) targeting a variety of subunits were screened to choose the antibody with the most efficient capture (Table S1). Before each capture, lysates were precleared with 25 μL of equilibrated Protein A Sepharose slurry (GE Healthcare) for 3 h. For each antibody, 25 μl was incubated with 300 μl of cell lysate at 4 °C overnight, after which 25 μl of Protein A Sepharose slurry was added to the mixture. Following a 3-h incubation at 4 °C, the IP reaction was centrifuged for 3 min at 1000 rpm, followed by a wash with 500 mM NaCl, two washes with lysis buffer, then one wash with 10 mM Hepes. Beads were eluted using 0.2% trifluoroacetic acid in 20% acetonitrile. Aliquots of lysate input, IP supernatant fraction (unbound), and eluate were saved for confirmation by Western blotting. To generate a negative control for affinity capture, the chosen optimal antibody (CST 4257S) was preincubated with its corresponding blocking peptide at 1:1 v/v ratio for 2 h at room temperature, and an IP was carried out as described earlier. Validation studies followed the same protocol, along with a complimentary ZC3H14 IP carried out using ab169061 (Abcam), which only recognizes isoform 1.

### iTRAQ labeling and mass spectrometry

The preparation and analysis of immunoaffinity preparations followed published methods (93, 94). Seven (three biological replicates of CFM-treated, three biological replicates of ACM-treated, one negative control), affinity capture eluates were dried in a centrifugal evaporator to remove the acetonitrile and trifluoroacetic acid. Next, protein samples were denatured with 9 M urea at room temperature. Subsequently, 1 M tetraethylammonium bicarbonate was added to adjust the pH to 8.0, followed by cysteine reduction for 30 min at 60 °C at 5 mM tris(2-carboxyethyl) phosphine. Protein sulfhydryl groups were then alkylated for 1 h at room temperature in the presence of 10 mM 4-vinylpyiridine. The urea concentration was decreased below 1.5 M by dilution with 500 mM tetraethylammonium bicarbonate, then the protein was digested with porcine trypsin (Thermo Fisher Scientific) at 37 °C overnight. Isobaric labeling of the tryptic digests with an iTRAQ 8plex reagent set (Sciex) was performed according to the manufacturer's protocol. The iTRAQ modified digests were pooled, and the mixture was purified in parallel on a C18 Bond Elut OMIX column (Agilent Technologies) at pH 2 and on a high pH reversed-phase peptide fractionation kit (Thermo Fisher) from which fractions were collected at 12.5%, 17.5%, 22.5%, and 50% acetonitrile. All purified samples were dried in a centrifugal evaporator, reconstituted in water containing 5% acetonitrile and 0.1% formic acid, then analyzed on a Tribrid Orbitrap Fusion (Thermo Fisher Scientific). Two technical replicates of the pH 2 C18 preparation and one technical replicate of each high pH reversed phase fraction were run. Peptide sequence information was generated from MS and MS2 scans while quantitative iTRAQ reporter ion information was generated from MS3 scans of the 10 most abundant product ions in each MS2 spectrum. MS, MS2, and MS3 scans were acquired in Orbitrap, linear ion trap, and Orbitrap mass analyzers, respectively. The time allowed between MS scans was fixed to a maximum of 3 s, during which time the maximum number of MS2 and MS3 spectra were collected. Mass spectra were collected during 260-min liquid chromatography runs having an acetonitrile content increasing from 0% to 30% in 180 min, then to 100% in 60 min, then remaining at 100% for 20 min. The 25-cm long, 75-μm inner-diameter analytical column contained Acclaim PepMap RSLC C18 particles of 2 μm diameter with 100-Å pores and was operated at 300 nl/min. The mass spectrometry proteomics data have been deposited to the ProteomeXchange Consortium via the PRIDE partner repository (95) with the dataset identifier PXD022200.

### Interactomics

Protein identification and quantification was conducted with Mascot (version 2.4; Matrix Science Ltd, London, UK) and Sequest HT search engines within Proteome Discoverer software (version 1.4; Thermo Fisher Scientific) as well as PEAKS studio 8.5 (Bioinformatics Solutions Incorporated, Waterloo, ON, Canada) using the mouse international protein index (IPI) database (version 3.87, 59,534 entries). The search was constrained to peptides with fully tryptic cleavage and up to two missed cleavages per peptide, fixed iTRAQ 8-plex modification at amino-termini and lysine residues, fixed pyridylethylation at cysteine residues, precursor mass deviation within 20 ppm, and product ion mass deviation up to 0.4 Da. Deamidation at asparagine or glutamine, oxidation at methionine, and phosphorylation at serine, threonine, or tyrosine were allowed variable modifications. The false discovery rate was fixed at 0.05 and determined by Percolator with validation based on q-value in Proteome Discoverer or with the PEAKS algorithm. iTRAQ reporter ion signals were extracted from MS3 spectra in the QUANTITATION algorithm of PEAKS and the Reporter Ions Quantifier in Proteome Discoverer. Reporter Ions Quantifier integration tolerance was 20 ppm, and integration was based on the most confident centroid. Included in protein quantification were PSMs scoring above false discovery rate cutoffs. Protein levels were normalized to one ACM replicate (ACM3), which was arbitrarily chosen as an internal reference. To determine the extent to which PI3K interactions changed as a result of ACM treatment, the data was filtered over three main stages: (1) Proteins with negative control:ACM3 ratios >0.4 were filtered out to eliminate nonspecific binders. (2) Candidate interactors were those proteins having an ACM1:ACM3 or ACM2:ACM3 ratio between 0.65 and 1.35, an indicator of high reproducibility between ACM triplicates. (3) Among highly reproducible candidates, PI3K interactor proteins with a CFM:ACM of <0.65 were considered ACM-induced interactors and those of >1.35 were considered to have been dissociated by ACM (Table S-2). Known interactors were identified using the STRING database (http://string-db.org) (96).

### siRNA transfection

A previously validated siRNA sequence was used for ZC3H14 knockdown (46) (TGACTGACCTGAGTGTGGCACAGAA), compared with scrambled control (Stealth RNAi, Thermo) in cultured Ht22 cells, according to the manufacturer's directions. Briefly, cells were plated at a density of 1.5 × 10^5^ cells per well in 6-well plates in 10% FBS DMEM. Each well received 4 μl of Lipofectamine 2000 (Thermo), diluted in 150 μl of Optimem media (Thermo); siRNA was also diluted in 150 μl of Optimem media. Lipofectamine 2000-siRNA complexes were then combined with 1200 μl of serum-free DMEM and applied to cells for 6 h. The medium was then replaced by fresh 10% FBS DMEM. ZC3H14 knockdown was evaluated by immunoblotting 48 h following transfection.

### PLA assay

A proximity ligation assay was performed for PI3K and ZC3H14 probes according to the manufacturer's directions (Sigma). Briefly, rat RGCs were plated at 8000 cells per well on 96-well plates. Cells were fixed in 4% paraformaldehyde, washed 2× in PBS and 1× in PBST, then blocked for 1 h in donkey serum and incubated with primary antibodies overnight at 4 °C. The next morning, cells were washed and incubated with 30 μl diluted Duolink probes and incubated for 1 h at 37 °C. The prepared ligase mixture was then added to washed cells and incubated for 30 min at 37 °C, followed by polymerase mixture for 100 min at 37 °C. Finally, cells were washed, with DAPI added to the final wash. Negative controls consisted of wells missing one or both primary antibodies. PLA images were obtained using a Nikon Confocal Microscope to detect a neu-N neuronal marker antibody staining at 488 nm, and the PLA signal at 561 nm, with the same settings across groups. Nikon NIS-Elements Advanced Research software, v 4.51, was used to acquire corresponding images using the 488-nm signal to threshold for neurons and measure mean intensity of the 561-nm PLA signal per cell.

### Statistics

For all experiments, n refers to the number of biological replicates or animals. Data were assessed for normality and statistical analyses were performed by *t* test (Fig. 1, *C* and *F*) or one-way ANOVA with either Dunnet (Figs. 1*D* and 4*B*) or Tukey (Fig. 5*D*) post hoc analyses. Figure 4*D* was analyzed by two-way ANOVA with Tukey multiple comparisons test. Corresponding *p* values are reported in each figure or legend.

## Data availability

All data are included in the article or are available upon request. The mass spectrometry proteomics data have been deposited to the ProteomeXchange Consortium via the PRIDE partner repository (96) with the dataset identifier PXD022200.

## Conflict of interest

The authors declare that they have no conflicts of interest with the contents of this article.
