# Supplementary material for: An endogenous PI3K interactome promoting astrocyte-mediated neuroprotection identifies a novel association with RNA-binding protein ZC3H14
Source: J Biol Chem. 2020 Dec 3;296:100118. doi: 10.1074/jbc.RA120.015389 (PMC7948738; doi:10.1074/jbc.RA120.015389)
Supplement: Figures S1 to S7 [file mmc1.pdf]

Figure S-1

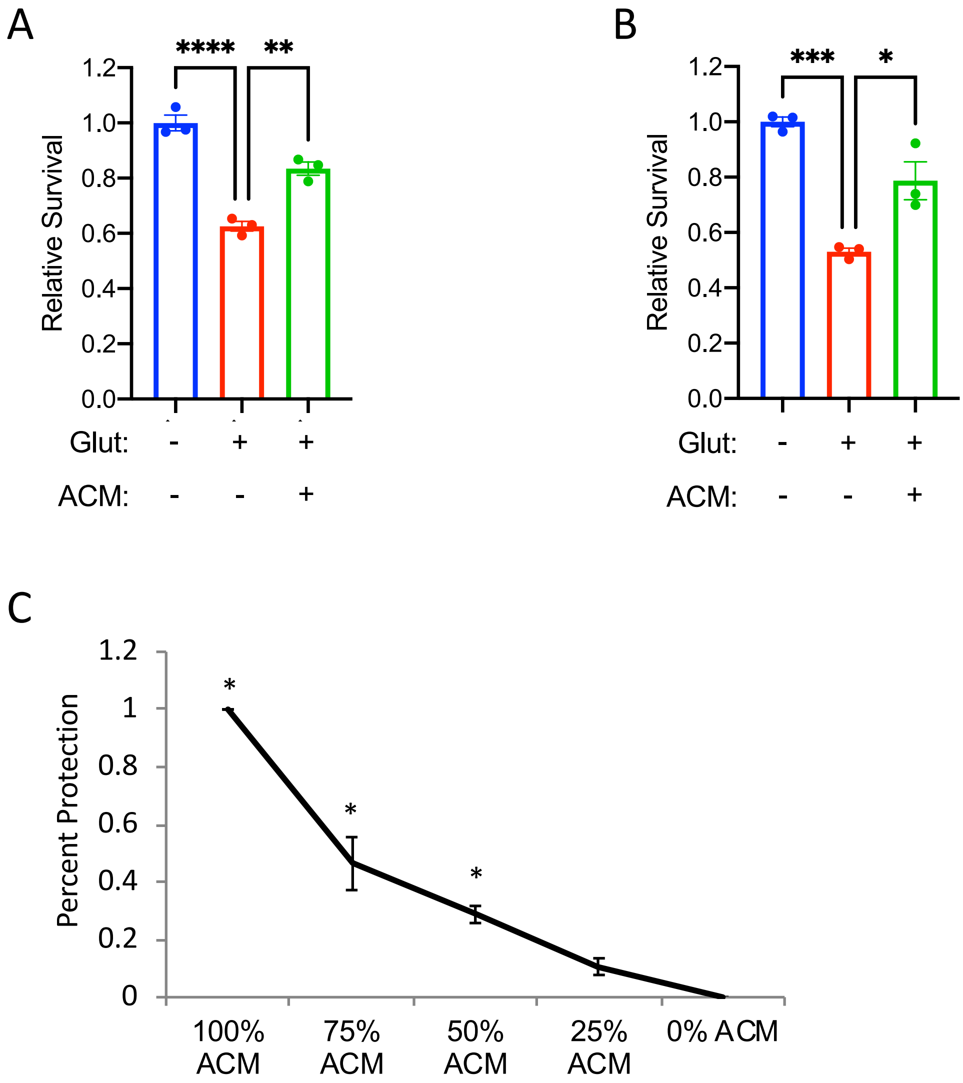

Figure S-1. Optimization of neuronal ACM protection screening assay. A) Example of an Ht22 XTT cell viability assay injured with 5mM glutamate and ACM induced protection (****p<0.0001, **p=0.002). B) For comparison, similar protection results are seen with a live cell presto blue assay (***p=0.0005, *p=0.01). C) Dilution curve of ACM protection in Ht22 cells against 5 mM glutamate oxidative injury. Partial ACM activity is maintained when diluted to 25% of volume, beyond which the neuroprotective activity is lost (n=3, *p<0.05).

Figure S-2

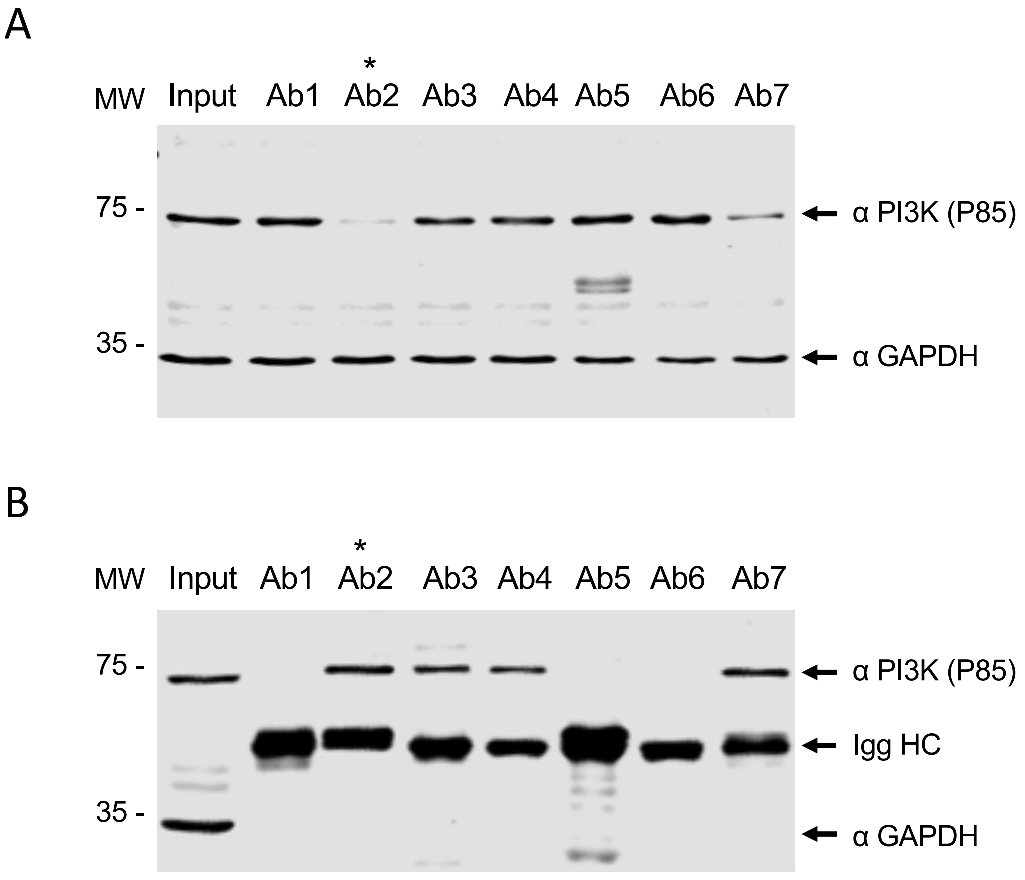

Figure S-2. PI3K Immunoprecipitation antibody screen. A monoclonal PI3K antibody panel was screened to identify an optimal reagent antibody for affinity capture. (A) A blot comparing unbound fractions shows that Ab2 (*) depleted most of the target protein as shown by the reduced PI3K band compared to GAPDH (arrows). (B) A blot comparing eluted proteins shows that four antibodies captured PI3K effectively (A2, Ab3, Ab4, and Ab7) with Ab2 demonstrating strongest captured PI3K band (arrow). Note: in eluates GAPDH is only present in the input lane (arrow)

Figure S-3

A

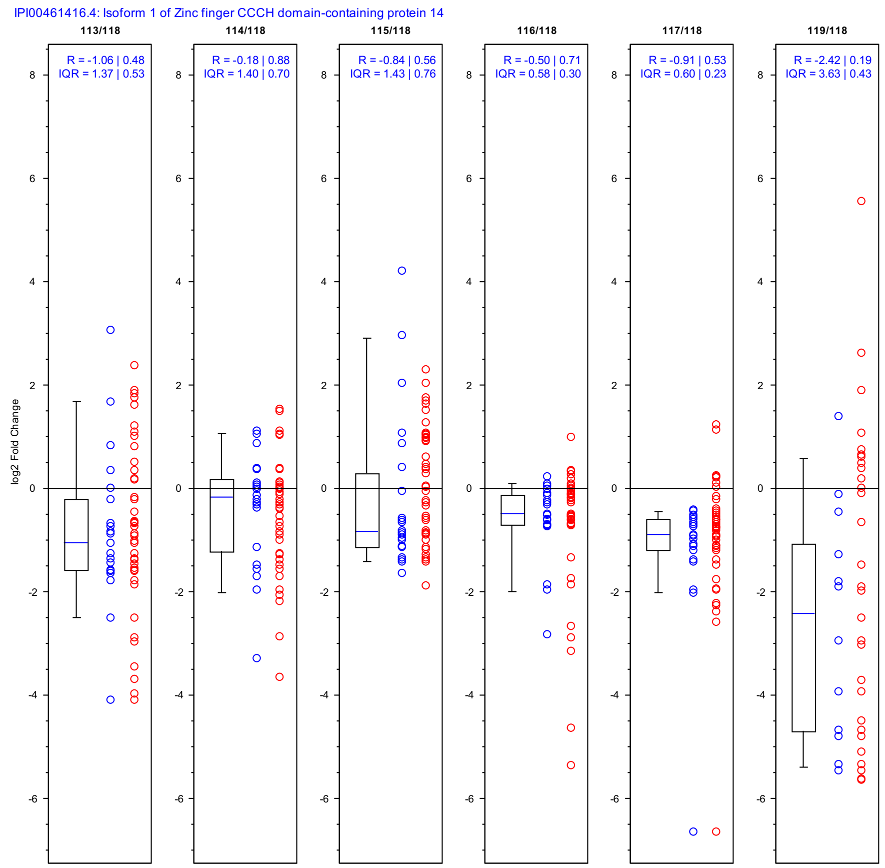
IPI00461416.4: Isoform 1 of Zinc finger CCCH domain-containing protein 14
113/118
114/118
115/118
116/118
117/118
119/118
R = -1.06 | 0.48
IQR = 1.37 | 0.53
R = -0.18 | 0.88
IQR = 1.40 | 0.70
R = -0.84 | 0.56
IQR = 1.43 | 0.76
R = -0.50 | 0.71
IQR = 0.58 | 0.30
R = -0.91 | 0.53
IQR = 0.60 | 0.23
R = -2.42 | 0.19
IQR = 3.63 | 0.43
log2 Fold Change

B

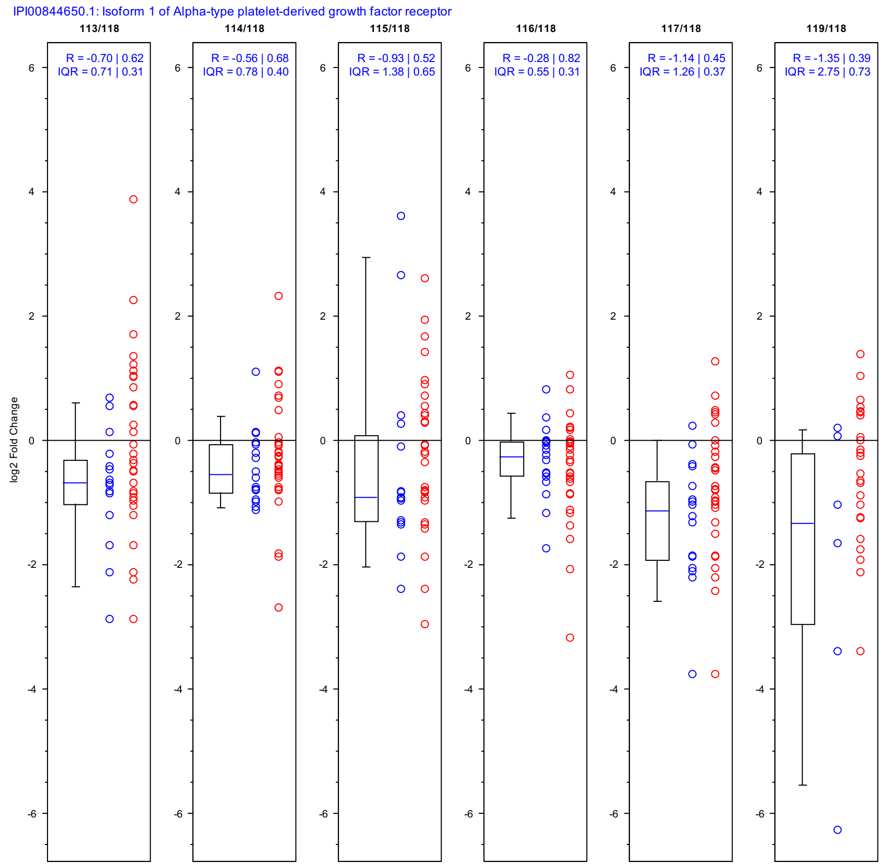
IPI00844650.1: Isoform 1 of Alpha-type platelet-derived growth factor receptor
113/118
114/118
115/118
116/118
117/118
119/118
R = -0.70 | 0.62
IQR = 0.71 | 0.31
R = -0.56 | 0.68
IQR = 0.78 | 0.40
R = -0.93 | 0.52
IQR = 1.38 | 0.65
R = -0.28 | 0.82
IQR = 0.55 | 0.31
R = -1.14 | 0.45
IQR = 1.26 | 0.37
R = -1.35 | 0.39
IQR = 2.75 | 0.73
log2 Fold Change

Figure S-3 (*cont*)

C

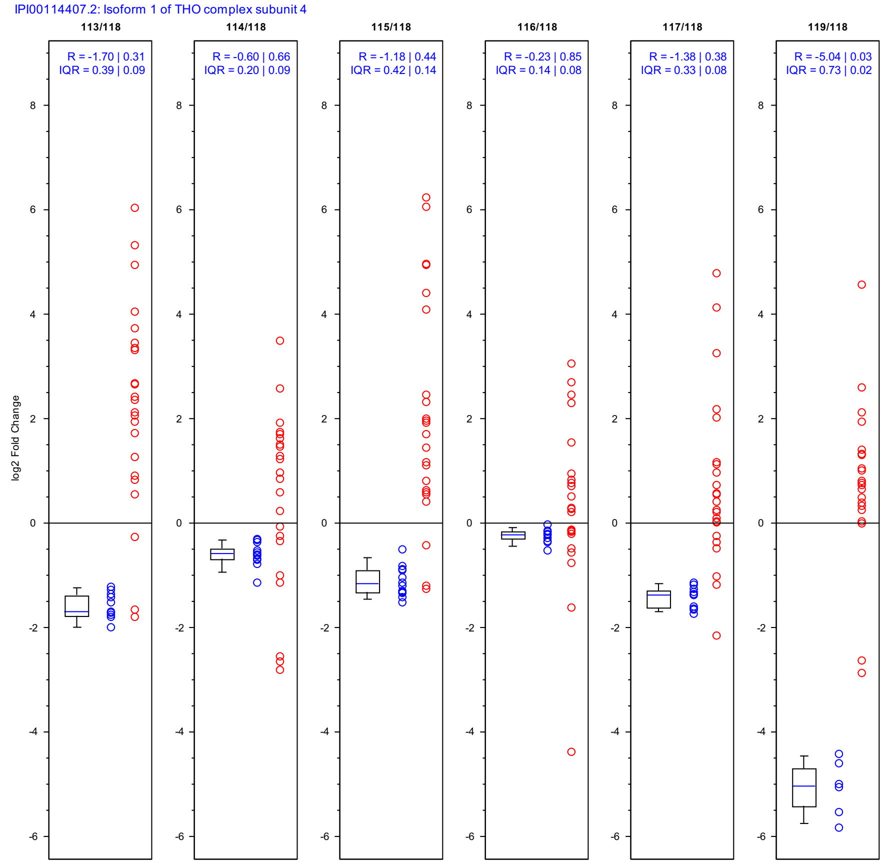

D

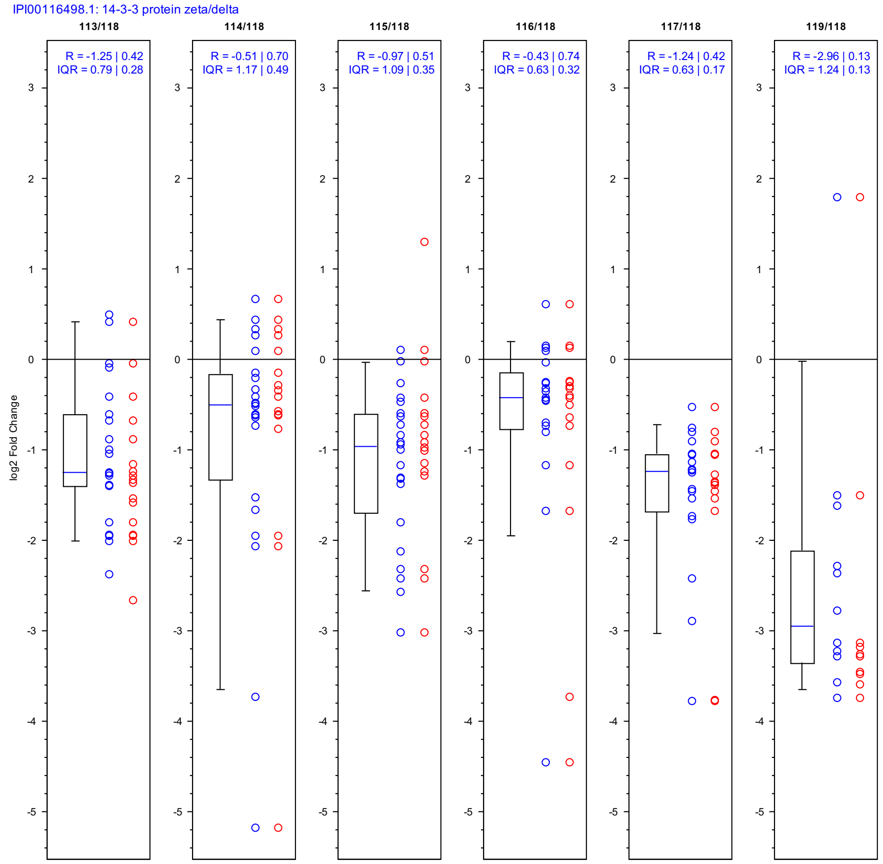

Figure S-3 (*cont2*)

E

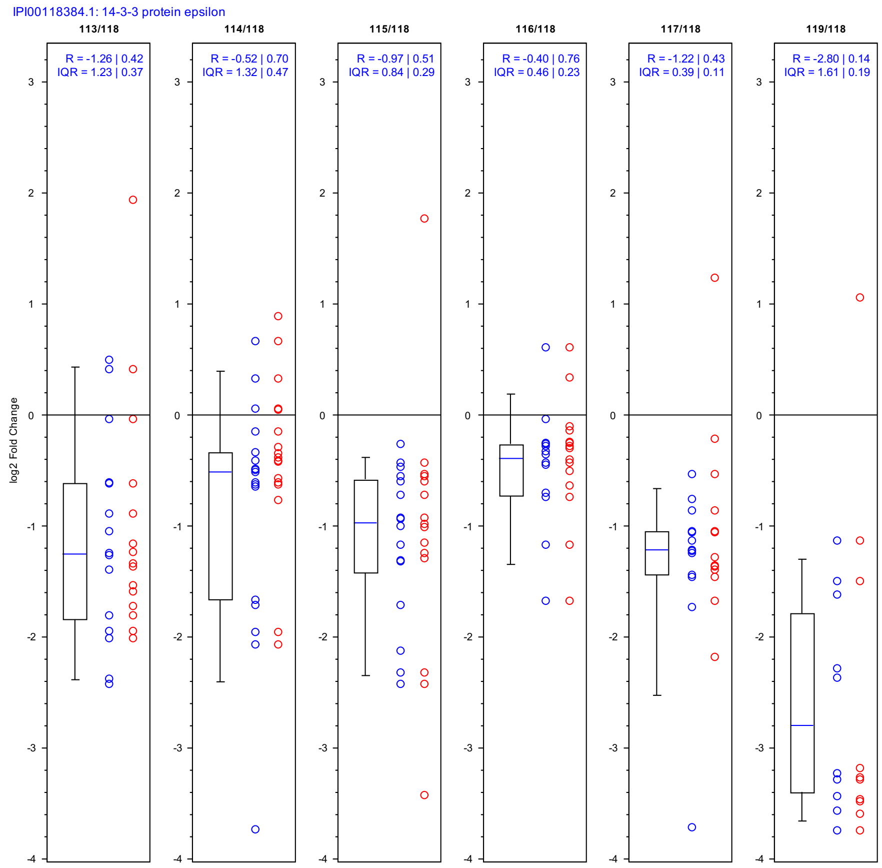

Figure S-3. Box plot depicting iTRAQ quantification of ACM-regulated PI3K binders relative to ACM3, including (A) Isoform 1 of Zinc finger CCCH domain-containing protein 14, (B) Isoform 1 of Alpha-type platelet-derived growth factor receptor, (C) Isoform 1 of THO complex subunit 4, (D) 14-3-3 protein zeta/delta, and (E) 14-3-3 protein epsilon. The computed Median peptide ratios and Inter Quartile Ranges (IQR) are shown above the graph.

Figure S-4

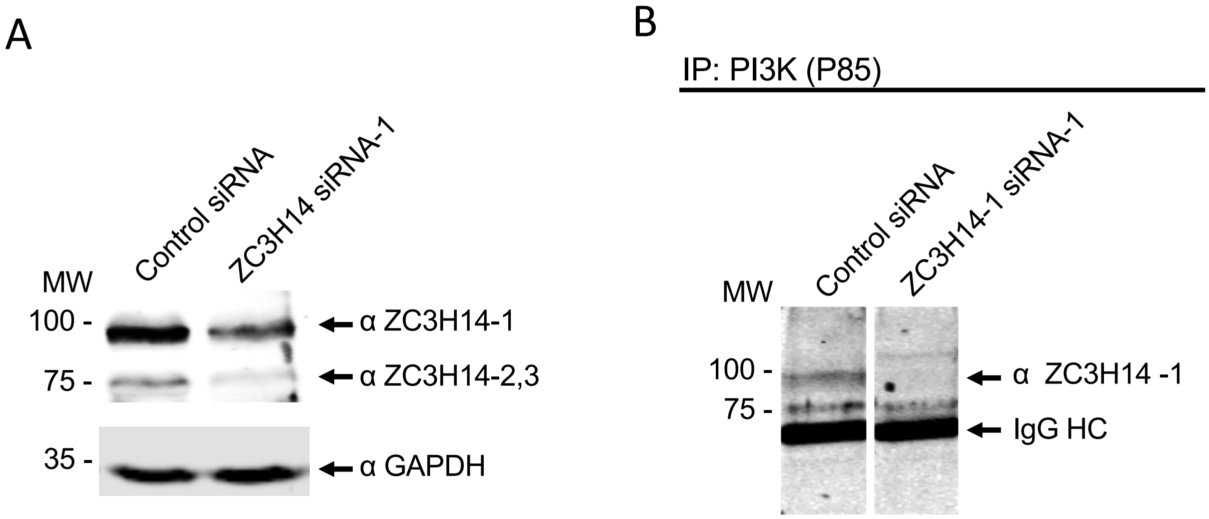

Figure S-4. Effective ZC3H14-1 knock down. A) A western blot of Ht22 cell lysates showing the effectiveness of ZC3H14 siRNA to reduce levels of the protein compared to a scrambled control siRNA (Control). The ZC3H14-1 sequence reduces ZC3H14-1 and ZC3H14-2,3 isoforms by 64% and 78%, respectively, when normalized to GAPDH (arrows). B) A co-immunoprecipitation with PI3K (P85) capture demonstrating that the ZC3H14-1 band is reduced with ZC3H14 siRNA-1 knockdown, in comparison to control siRNA (arrow, lanes are from same gel).

Figure S-5

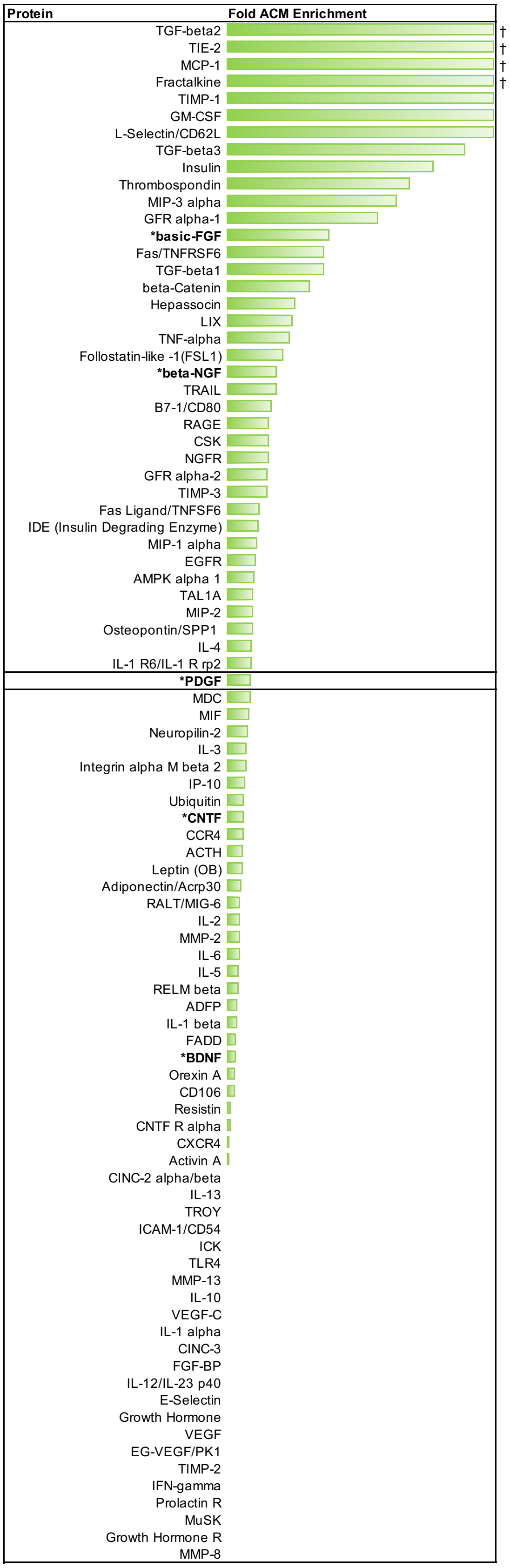

Figure S-5. Protein enrichment in ACM. Relative fold enrichment of ACM over CFM concentrations for a multiplex panel of 90 cytokines, growth factors and other molecules (n=3). PDGF is outlined with a black border. (*denotes RTK binding growth factors with reported neuroprotective activities, † indicates factors not detected in CFM resulting in infinite enrichment.)

Figure S-6

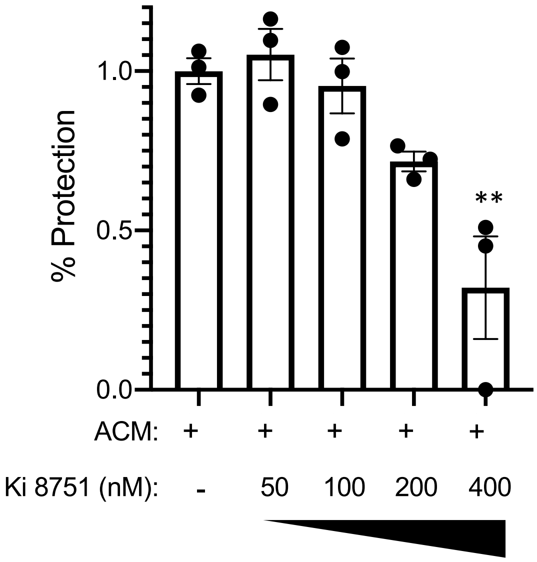

Figure S-6. Inhibition of PDGFRA blocks ACM neuroprotection. Inhibition of PDGFRA signaling with the kinase inhibitor Ki 8751partially blocks ACM induced protection of Ht22 cells from excitotoxic injury in a dose dependent manner (n=3, **p=0.002, bars are SE).

Figure S-7

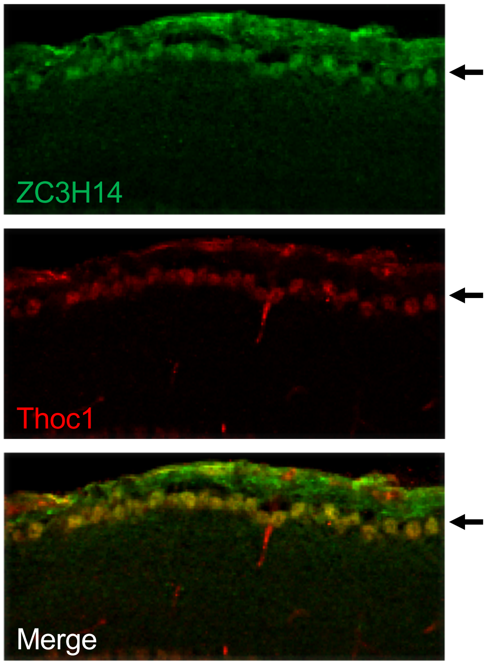

Figure S-7. ZC3H14 and Thoc1 colocalize in the inner retina. Mouse retinal sections probed for ZC3H14 (green) and Thoc1 (red), showing strong colocalization in the ganglion cell layer (arrow). (Scale bar indicates 40 μM).
