# Supplementary material for: An endogenous PI3K interactome promoting astrocyte-mediated neuroprotection identifies a novel association with RNA-binding protein ZC3H14
Source: J Biol Chem. 2020 Dec 3;296:100118. doi: 10.1074/jbc.RA120.015389 (PMC7948738; doi:10.1074/jbc.RA120.015389)
Supplement: Table S1 and S2 [file mmc2.docx]

**Supporting Information**

**Table S-1.** PI3K antibodies tested in immunoprecipitation screen, along with molecular weight of target peptide, and corresponding IgG isotype

| Ab # | Antibody description and catalogue number | Size (kD) | IgG isotype |
| --- | --- | --- | --- |
| Ab 1 | Phospho-PI3 Kinase p85 (Tyr458)/p55 (Tyr199) Antibody 4228 | 60 and 85 | Rabbit IgG |
| Ab 2 | PI3 Kinase p85 (19H8) Rabbit mAb 4257 | 85 | Rabbit IgG |
| Ab 3 | PI3 Kinase p110α (C73F8) Rabbit mAb 4249 | 110 | Rabbit IgG |
| Ab 4 | PI3 Kinase p110β (C33D4) Rabbit mAb 3011 | 110 | Rabbit IgG |
| Ab 5 | PI3 Kinase Class III (D4E2) Rabbit mAb 3358 | 100 | Rabbit IgG |
| Ab 6 | PI3 Kinase p110γ (D55D5) Rabbit mAb 5405 | 110 | Rabbit IgG |
| Ab 7 | PI3 Kinase p85 Antibody 4292 | 85 | Rabbit IgG |

**Table S-2.** Thresholds applied to complete data set from PI3KR1 interactome to identify PI3KR1 interactors and media composition-dependent PI3KR1 interactors

| Variable | CFM1/ACM3 count | CFM2/ACM3 count | CFM3/ACM3 count | ACM1/ACM3 count | ACM2/ACM3 count | CFM1/ACM3 (113/118) | CFM2/ACM3 (115/118) | CFM3/ACM3  (117/118) | ACM1/ACM3 (114/118) | ACM2/ACM3 (116/118) | Negative/  ACM3 (119/118) |
| --- | --- | --- | --- | --- | --- | --- | --- | --- | --- | --- | --- |
| Filter | 2> | | | | | 1.35<n<0.65 | | | 1.35>n>0.65 | | <0.4 |
| Purpose | Threshold for accuracy of protein quantification | | | | | Threshold for media composition-dependent PI3KR1 interactors | | | Threshold for accuracy of protein quantification | | Threshold for PI3KR1 binding |
